# Sulfonylurea use and the risk of hospital readmission in patients with type 2 diabetes

**DOI:** 10.1186/s12902-016-0084-z

**Published:** 2016-01-20

**Authors:** Pamela C. Heaton, Vibha C. A. Desai, Christina M. L. Kelton, Swapnil N. Rajpathak

**Affiliations:** Pharmacy Practice and Administrative Sciences, James L. Winkle College of Pharmacy, University of Cincinnati Academic Health Center, Cincinnati, OH 45267 USA; HealthCore, Andover, MA 01810 USA; Carl H. Lindner College of Business, University of Cincinnati, 414 Lindner Hall, 2925 Campus Green Drive, Cincinnati, OH 45221 USA; US Outcomes Research, Merck & Co., Inc., Whitehouse Station, NJ 08889 USA

**Keywords:** Sulfonylureas, Oral antihyperglycemic agents, Type 2 diabetes, Hospital readmissions

## Abstract

**Background:**

Hospital inpatient care for patients with diabetes was estimated to cost $76 billion in 2012. Substantial expense resulted from those patients having multiple hospitalizations. The objective was to compare the risk for diabetes–related hospital readmission in patients with type 2 diabetes treated with sulfonylureas (SUs) compared to those treated with other oral antihyperglycemic agents (AHAs).

**Methods:**

A retrospective cohort analysis was conducted using two-year panels, from 1999 to 2010, from the Medical Expenditure Panel Survey. The study included patients with type 2 diabetes taking an oral AHA who experienced a diabetes-related hospitalization. A Cox proportional hazard regression predicting time to readmission was used to estimate and compare the risks of readmission for SU-monotherapy versus other-AHA-monotherapy patients. Covariates included age, gender, marital status, cardiovascular disease, kidney disease, and eye disease, along with a propensity score to control for selection bias. The lack of clinical data on disease severity and progression limited our ability to estimate causal relationships between drug use and risk of hospital readmission.

**Results:**

From 1999 to 2010, an estimated 13.5 million patients experienced a diabetes-related hospital admission and subsequent AHA treatment. While 23.2 % (n = 746,579) of patients in the SU monotherapy cohort had a readmission, only 16.1 % (n = 881,984) in the other-AHA monotherapy group were readmitted. Average readmission expenditure for readmitted SU users (in 2010 dollars) was $11,148 (±$1,558) compared to $7,673 (±$763) for users of other oral AHAs. The estimated readmission hazard ratio was 1.29 (95 % CI: 1.01–1.65; p-value = 0.04) for SU monotherapy users. If a patient’s first hospital admission was during the time period 2008–2010, a readmission was significantly less likely (HR 0.49, 95 % CI: 0.31–0.78; *p* = 0.003) relative to 2004–2007.

**Conclusions:**

Among patients with type 2 diabetes, SU use was associated with an approximately 30 % increased risk for readmission compared to other-AHA use, while each readmission for an SU user cost on average 45 % more than one for an other-AHA patient. Because of the rapidly rising prevalence of diabetes in the U.S. and the large number of patients with prediabetes, preventing hospital readmissions will continue to be an important cost-saving strategy in the future.

## Background

In the United States in 2014, 29.1 million people, 9.3 % of the population, were estimated to have diabetes [1]. Incidence was recently estimated at 11.5 per 1,000 adults [2]. The disease cost $245 billion in 2012, including $176 billion in direct medical costs and $69 billion in reduced productivity [3], conservative estimates partly because of the inability to account for undiagnosed persons with diabetes, estimated at 6.3 million in 2007 [4] and 8.1 million in 2014 [1]. Moreover, omitted from the estimated cost burden was the value of care provided by unpaid caregivers as well as reduced quality of life for patients and caregivers both [3].

The largest component of medical expenditures for patients with diabetes in 2012 was hospital inpatient care, estimated at $76 billion, and there were 26,383 hospital days in 2012 attributable to diabetes [3]. Much of this hospital expense and time resulted from patients having multiple hospitalizations. According to Jiang and colleagues, among patients with diabetes who had been hospitalized, 30 % had two or more stays accounting for 50 % of total hospitalizations and hospital costs [5]. In another study, Jiang and colleagues found that 21 % of patients with diabetes were readmitted to the hospital within 30 days of discharge, and 45 % were readmitted within 45 days [6]. Both studies relied on commercial claims databases. To date, however, no nationally representative, multi-payer estimates of the total number of patients with diabetes undergoing multiple hospitalizations exist.

Oral antihyperglycemic agents (AHAs), including biguanides, thiazolidinediones (TZDs), alpha-glucosidase inhibitors, meglitinides, dipeptidyl peptidase 4 (DPP-4) inhibitors, and sulfonylureas (SUs), can be used either individually or in combination to treat diabetes. Because many hospitalizations of patients with diabetes result from hypoglycemia [7, 8] and because SUs, through excessive insulin production and release, lead to episodes of hypoglycemia [9, 10], it is reasonable to hypothesize that patients who are on an SU are at an increased risk of hospitalization relative to patients who take other AHAs. In two previous studies that have attempted to predict diabetes-related hospitalizations based on medication use, results were mixed. Quilliam and colleagues reported that, relative to no use of SUs, continuous and intermittent use of SUs was associated with a two-fold increase in the number of hypoglycemic-related hospitalizations [11]. However, using a different database and methodology, another study concluded that SU use was not predictive of hypoglycemic-related emergency-department and outpatient visits [12]. To date, however, no study has evaluated whether SU use is related to a higher risk for hospital readmission.

Therefore, with two important gaps in the literature, our objective was to estimate, using an all-payer, nationally representative database, time to hospital readmissions for patients treated, after their first hospitalization, with SUs versus other oral AHAs. Secondary objectives were to estimate costs for the readmissions and to document changes that occurred in both readmission rates and pharmacotherapy for patients with type 2 diabetes over the decade of the 2000s.

## Methods

### Data

A retrospective cohort analysis was conducted using longitudinal data (two-year panels from 1999 to 2010) from the Medical Expenditure Panel Survey (MEPS), a nationally representative and publicly available survey of healthcare utilization and costs in the United States. In the MEPS panels, sampled households participated in five rounds of interviews over the course of two years. Survey responses were then validated by the household’s healthcare providers, such as pharmacists, physicians, and hospitals. All healthcare expenditures for the households and sources of payment, over the two-year period, were also collected by the MEPS. Partly because of supplemental data obtained for households with members suffering from diabetes, the MEPS has been used extensively to study diabetes [13, 14], including drug utilization by those with diabetes [15].

The MEPS database can be used to generate national estimates of healthcare utilization and cost if appropriate weights obtained from stratification and clustering variables are applied. The MEPS includes both general patient weights and patient-with-diabetes-specific weights. When available, the latter were used in this study; when not, the general weights were applied. The consistency over time in the MEPS design allows for pooling data over multiple panels, often necessary in order to obtain reliable national estimates based on high enough (>100) raw cell counts [16].

### Patient selection

Patients with a diabetes-related hospital admission during their two-year panel were identified from the inpatient and emergency-department MEPS files by having one of the ICD-9 diagnosis codes in Appendix 1 Table 4 as one of their listed (not necessarily primary) diagnoses [6]. Among this group of patients, only those receiving an oral AHA post-hospitalization, but before any diabetes-related readmission if one occurred, were considered for further study. Patients receiving insulin, any time prior to their second hospital admission, identified by the level-2 therapeutic class code 215 from the prescribed-medicines file in the MEPS, were excluded. Patients who were diagnosed as pregnant (ICD-9 code V22.x) were also excluded.

### Hospital readmission

Patients with a hospital readmission were identified as having a subsequent hospital admission for a diabetes-related condition within one year of the first admission. For patients with no readmission, we examined claims for three rounds following the round of the first admission or until the end of the two-year panel, whichever occurred first.

### Cohort identification

Depending on their medication use (see Appendix 1 Table 5 for level-2 therapeutic classification codes for drugs) following their first hospital admission, patients were categorized as follows: those on SU monotherapy (SU cohort); those on combination therapy that included an SU (SU+ cohort); those on monotherapy with a non-SU AHA (noSU cohort); and those on combination therapy without an SU (noSU+ cohort).

### Statistical analysis

Although descriptive readmission and cost statistics were obtained for all four patient cohorts, all other analyses were performed using monotherapy patients (SU and noSU groups) in order to preserve a clean comparison between the SU and other drug classes. The outcome variable, time to readmission, was computed as the number of days between the first and second hospitalization.

Covariates were all baseline patient characteristics potentially associated with initial drug assignment, as well as with time to readmission. They included demographic characteristics (age, gender, race, geographic location, and marital status); insurance coverage; period of first hospital admission (to detect any potential trend over time); comorbidities (cardiovascular disease, renal disease, and eye disease); medical care received (number of HbA1c tests and difficulty in getting care); and disease severity (perceived health status, perceived mental health status, and limitations in physical functioning).

A logistic regression model, with assignment to the SU cohort as the dependent variable and the covariates as independent variables, was estimated to obtain propensity scores for SU use. To determine whether the propensity score achieved balance across the covariates, each covariate was regressed, by way of an appropriate dependent-variable specification (logit, multinomial logit, or ordered logit), on the treatment (SU versus noSU) and the patient’s propensity score [17, 18]. Balance was determined based on a p-value for the estimated treatment coefficient of > 0.05. Because balance for all covariates was indeed achieved according to the 0.05 criterion following this procedure, no additional statistical measures were taken to guard against selection bias.

Kaplan-Meier survival curves were generated from the time-to-readmission data for each of the study arms. Then, using a Cox proportional hazard model, time to readmission was regressed on study-arm assignment, a subset of the baseline patient characteristics (for parsimony, not all variables were included), and the propensity scores, following a covariate-adjustment procedure from the propensity-score literature [19]. Patients without a readmission experienced a censoring endpoint, which was either one year from the date of first hospital admission or the end of the MEPS panel, whichever occurred first.

Additional statistical tests were run to ensure correct model specification. All analyses were conducted using SAS version 9.2 and R version 2.14.2. With the exceptions of the Kaplan-Meier curves and the *t*-test for equality of mean age between cohorts, which could not be accomplished in SAS from weighted survey data, all analyses, including the propensity-score analysis, used population-weighted MEPS data, necessary to obtain unbiased treatment effect estimates that are generalizable to the original survey target population [20]. Because of the public nature of the MEPS database, the research did not require review and approval by the Institutional Review Board at the University of Cincinnati.

## Results

From 1999 to 2010, an estimated 19.0 million patients experienced a diabetes-related hospital admission and subsequent AHA treatment. After exclusions for insulin use (5.5 million patients) and pregnancy (9,937 patients), 13.5 million patients remained in the study. Of these patients, 7.87 million patients were on SU therapy (SU or SU+), and the remaining 5.67 million were on non-SU oral agents (noSU and noSU+) as shown in Table 1.

**Table 1:** Hospital Readmission Rates and Average Readmission Costs for Medication-Based Patient Cohorts: 1999–2010. The readmission percentage is found by dividing the number readmitted to the hospital by the total number of patients who experienced an initial diabetes-related hospitalization. Readmission cost is expressed in 2010 U.S. dollars

Patient Cohort	Total number (SE)	Number not readmitted (SE)	Number readmitted (SE)	Readmission percentage	Mean readmission cost (SE)
All Patients	13,537,803 (519,634)	10,959,266 (451,218)	2,578,538 (198,384)	19.1	$8,814 ($580)
All SU Patients ^a^	7,871,912 (368,351)	6,204,869 (326,273)	1,667,043 (153,735)	21.2	$9,204 ($769)
SU (SU monotherapy) ^b^	3,217,089 (235,951)	2,470,510 (194,884)	746,579 (106,554)	23.2	$11,148 ($1,558)
SU+ (other AHA)	4,654,823 (274,495)	3,734,359 (247,494)	920,464 (113,487)	19.8	$7,624 ($412)
All Other Oral AHA Patients ^a^	5,665,891 (291,467)	4,754,397 (256,821)	911,495 (108,690)	16.1	$8,098 ($737)
noSU (monotherapy with non-SU AHA) ^b^	5,488,379 (288,154)	4,606,396 (254,357)	881,984 (107,289)	16.1	$7,673 ($763)
noSU+ (> 1 non-SU AHA)	177,512 (na)	148,001 (na)	29,511 (na)	16.6	$20,772 (na)

*SE* standard error

*na* not available due to too low raw cell count

*SU* sulfonylurea

*AHA* antihyperglycemic agent

^a^ In a comparison between all SU (SU and SU+) patients and all other oral AHA patients, the relevant p-value for readmission rates was 0.017

^b^ In a comparison between SU patients and noSU monotherapy patients, the relevant p-value for readmission rates was 0.003

Of those patients in the SU+ cohort, 3.0 million (64.6 %) were taking a biguanide concomitantly, while 1.1 million (24.7 %) were taking a TZD. There were very few (too few to obtain reliable national utilization estimates from the MEPS data) mentions of alpha-glucosidase inhibitors, meglitinides, or DPP-4 inhibitors among the cohort patients. Among patients in the noSU cohort, 3.9 million (70.7 %) were on a biguanide, while 1.4 million (24.8 %) were taking a TZD. Among patients in the noSU+ cohort, the only combination found in the MEPS was biguanide plus TZD.

Table 1 shows hospital readmission rates. There were in total 2.6 million (19.1 %) patients who experienced a readmission within one year of their first admission. Patients treated with an SU compared to those not treated with an SU experienced a significantly higher rate of readmission (21.2 % versus 16.1 %; *p* = 0.017). The difference in readmission rates was even greater for the SU (monotherapy) cohort alone versus the noSU (monotherapy) cohort (23.2 % versus 16.1; *p* = 0.003). In addition to the within-one-year readmission rates in Table 1, we also looked at the difference between the SU and noSU cohorts in 30-day readmission rates. For this comparison, the estimated difference was not significant at the 5 % significance level (10.2 % versus 7.3 %; *p* = 0.074).

In the last column of Table 1 are found mean costs of the readmissions. Along with higher readmission rates for SU users are higher expenditures for the readmissions, relative to users of other AHAs. Whereas the mean cost of a readmission for a patient in the SU cohort was, in 2010 dollars, $11,148 (±$1,558), the average was $7,673 (±$763) for a patient in the noSU cohort, representing a 45 % higher cost for an SU patient.

For all of the cohorts identified in Table 1, cardiovascular disease was the most frequently occurring diagnosis at readmission. The second and third most common diagnoses were hypoglycemia and diabetes, respectively. Renal disease and eye disease accounted for very low percentages of readmission diagnoses (< 10 % and < 1 %, respectively).

As seen in Table 2, patients in the SU cohort had some statistically significant (*p* < 0.05) differences in baseline characteristics relative to those in the noSU cohort. The SU cohort included older patients, with a mean age of 68.3 (95 % CI: 67.0–69.7), whereas the noSU patients were, on average, younger, with a mean age of 60.5 (95 % CI: 59.3–61.7). Whereas, for both sets of cohorts, the 2004–2007 time period for the first hospital admission saw the highest percentage of patients (45.86 % and 44.77 %, respectively), the percentage of SU users (31.46 %) was higher in the earliest time period than in the latest (22.68 %). The opposite was true for the noSU cohort (14.30 % in 1999–2003 and 40.93 % in 2008–2010). In the noSU cohort, the percentage of patients with cardiovascular disease at baseline (before or during the round of the first hospital admission) was significantly higher (65.17 %) than the percentage (52.72 %) of patients in the SU cohort. Moreover, SU users were more likely to have fair or poor perceived physical and mental health compared to users of other AHAs (54.95 % versus 43.62 % and 29.80 % versus 20.98 %, respectively). However, after propensity-score balancing, none of the estimated coefficients for treatment was found to be statistically significant, suggesting adequate control of selection bias by the propensity-score variable.

**Table 2:** Baseline characteristics for patients receiving antidiabetic monotherapy. Characteristics include demographic characteristics; insurance coverage; period of first hospital admission; comorbidities; medical care received; and disease severity. In order to achieve balance between the cohorts in characteristic distributions, propensity scores were estimated and used as a covariate in the Cox proportional hazard regression following a statistical check to see whether balance was achieved

Characteristic	All Patients (Percent)	SU Cohort (Percent)	noSU Cohort (Percent)	p-value ^a^	Adjusted p-value ^b^
Total	8,705,468	3,217,089	5,488,379		
Mean Age (95 % CI)	63.4 (62.5–64.4)	68.3 (67.0–69.7)	60.5 (59.3–61.7)	<0.0001	0.951
Male	3,738,700 (42.9)	1,514,251 (47.1)	2,224,449 (40.5)	0.093	0.985
Race					
White	6,859,909 (78.8)	2,525,415 (78.5)	4,346,796 (79.2)		
African American	1,305,820 (15.0)	466,478 (14.5)	856,187 (15.6)	0.784	0.912
Other	539,739 (6.2)	225,196 (7.0)	285,396 (5.2)		
Region					
Northeast	1,647,688 (18.9)	465,284 (14.5)	1,182,404 (21.5)		
Midwest	2,007,585 (23.1)	777,258 (24.2)	1,230,327 (22.4)	0.115	0.994
South	3,290,396 (37.8)	1,278,818 (39.8)	2,011,578 (36.7)		
West	1,534,591 (17.6)	526,591 (16.4)	1,008,000 (18.4)		
Married	4,530,506 (52.0)	1,578,073 (49.1)	2,952,432 (53.8)	0.193	0.992
Insurance status					
Public	3,410,507 (39.2)	1,455,344 (45.2)	1,955,164 (35.6)		
Private	4,442,060 (51.0)	1,457,269 (45.3)	2,984,792 (54.4)	0.0881	0.998
Other	223,194 (2.6)	136,278 (4.2)	86,916 (1.6)		
Uninsured	629,707 (7.2)	168,199 (5.2)	461,508 (8.4)		
Period of first hospital admission					
1999–2003	1,796,930 (20.6)	1,012,088 (31.5)	784,842 (14.3)		
2004–2007	3,932,720 (45.2)	1,475,349 (45.9)	2,457,371 (44.8)	<0.0001	0.937
2008–2010	2,975,819 (34.2)	729,652 (22.7)	2,246,166 (40.9)		
Cardiovascular disease	5,272,543 (60.6)	1,695,922 (52.7)	3,576,621 (65.2)	0.0017	0.966
Renal disease	823,544 (9.5)	354,703 (11.0)	468,840 (8.52)	0.298	0.979
Eye disease	1,454,104 (16.7)	547,914 (17.7)	886,190 (16.2)	0.624	0.995
Mean number of HbA1c tests (95 % CI)	3.5 (2.4–4.7)	3.1 (1.4–4.92)	3.8 (2.3–5.2)	0.602	0.443
Perceived health status					
Excellent/Very good	1,782,916 (20.5)	616,432 (19.2)	1,166,485 (21.3)		
Good	2,674,290 (30.7)	792,801 (24.6)	1,881,489 (34.3)	0.012	0.973
Fair/Poor	4,161,730 (47.8)	1,767,732 (55.0)	2,393,999 (43.6)		
Perceived mental health status					
Excellent/Very good	3,830,005 (44.0)	1,219,758 (37.9)	2,610,246(48.2)		
Good	2,678,886 (30.8)	998,646 (31.0)	1,680,241 (30.6)	0.017	0.919
Fair/Poor	2,110,045 (24.2)	958,560 (29.8)	1,150,485 (21.0)		
Physical limitations	4,119,188 (47.3)	1,634,470 (50.8)	2,484,718 (45.3)	0.153	0.991
Difficulty in getting care	4,541,484 (52.2)	1,554,111 (48.3)	2,987,374 (54.4)	0.107	0.989

*CI* confidence interval

^a^ p-value for the test of equality (t- or chi-squared) of percentage distributions between cohorts

^b^ p-value for the test of treatment effect on patient characteristic, controlling for patient propensity score

**Fig. 1:**
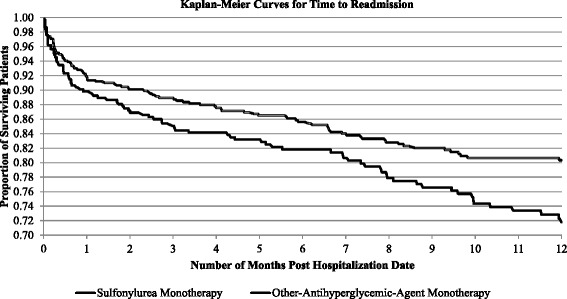
Kaplan-Meier curves for time to readmission within one year for patients receiving sulfonylurea monotherapy versus monotherapy with another oral antihyperglycemic agent. Each data point along the two curves represents the proportion of patients not readmitted to the hospital after a specified length of time following their first diabetes-related hospitalization. At the end of one year, approximately 72 % of patients taking a sulfonylurea remained out of the hospital, whereas over 80 % of patients not taking a sulfonylurea had not been rehospitalized

Figure 1 depicts the Kaplan-Meier survival curves for the two treatment groups, SU versus noSU. At 1 year, approximately 30 % of SU users had experienced a readmission, compared to 20 % of other-AHA users. Controlling for other factors affecting readmission, patients receiving SU monotherapy were significantly more likely to experience a readmission (HR 1.29, 95 % CI: 1.01–1.65; *p* = 0.042) than those in the noSU cohort (Table 3). Although not shown in Table 3, patients receiving SU monotherapy were more likely to experience a readmission (though not significantly so) compared to patients on metformin monotherapy (HR 1.18, 95 % CI: 0.93–1.52; *p* > 0.05). Other significant predictors of readmission were being single (HR 1.44, 95 % CI: 1.04–1.99; *p* = 0.030) and having eye disease (HR 1.45, 95 % CI: 1.06–2.00; *p* = 0.022). Moreover, if a patient’s first hospital admission was during the time period 2008–2010, a readmission was significantly less likely (HR 0.49, 95 % CI: 0.31–0.78, *p* = 0.003).

**Table 3:** Estimated hazard ratios from a Cox proportional hazard regression with time to hospital readmission as the dependent variable. After propensity-score adjustment, and controlling for demographic and other patient characteristics, patients on a sulfonylurea were 29 % more likely to be readmitted to the hospital than patients on another oral antihyperglycemic agent. Unmarried patients and patients with eye disease were also more likely to be readmitted

Independent variable ^a^	Hazard ratio	95 % confidence interval	p-value
Sulfonylurea	1.29	1.01–1.65	0.042
Age	1.01	0.98–1.03	0.749
Male	0.96	0.61–1.28	0.838
Race			
African American	0.89	0.66–1.28	0.512
Other	0.96	0.61–1.52	0.867
White	1.00		
Region			
Northeast	1.00	0.65–1.44	0.864
Midwest	0.86	0.62–1.18	0.349
West	1.28	0.94–1.74	0.110
South	1.00		
Unmarried	1.44	1.04–1.99	0.030
Period of first hospital admission			
1999–2003	0.69	0.37–1.28	0.235
2008–2010	0.49	0.31–0.78	0.003
2004–2007	1.00		
Cardiovascular disease	1.30	0.89–1.89	0.147
Renal disease	1.34	0.90–1.98	0.589
Eye disease	1.45	1.06–2.00	0.022
Perceived health status			
Good	0.76	0.49–1.18	0.221
Fair or poor	1.09	0.70–1.67	0.709
Excellent or very good	1.00		
Physical limitations	1.09	0.79–1.51	0.589

^a^ The patient propensity score was also included in the regression. Its estimated coefficient was not statistically significant (*p* > 0.100)

## Discussion

### Summary

This study documented, using a nationally representative database from 1999 to 2010, 2.6 million patients with type 2 diabetes and with multiple hospitalizations. Patients treated with SU monotherapy compared to those treated with other-AHA monotherapy experienced a significantly higher rate of readmission (23.2 % versus 16.1 %; *p* = 0.003). This study is the first to produce nationally representative readmission estimates and the first to study the relationship between pharmacotherapy and multiple admissions directly.

### Significance

Hospital readmissions are a major concern of healthcare providers and policymakers because they indicate deterioration, instead of improvement, of patients’ health. Whereas some readmissions have been associated with patient frailty and inevitable disease progression, many have been found to occur due to substandard care during the initial hospitalization, including poor resolution of the main problem, unstable therapy at discharge, and inadequate post-discharge care [21]. A section of the Patient Protection and Affordable Care Act of 2010, which allowed the Centers for Medicare & Medicaid Services to implement the Hospital Readmissions Reduction Program, has placed a spotlight on the need to reduce readmissions as a way to improve the quality of care and reduce costs across disease conditions, including diabetes [22]. To the extent that more effective medication management can occur pre- and/or post-discharge following an initial hospitalization, it may be possible to reduce the readmission rate for diabetes sufferers.

The number of people with diabetes and comorbid conditions is expected to increase precipitously in the near future. In fact, the $245 billion cost burden of diabetes in 2012 represented a 41 % increase over the $174 billion burden in 2007, in large part driven by a 27 % growth in diabetes prevalence over that five-year period [3]. According to the Centers for Disease Control and Prevention, an estimated 86 million adults are currently considered to have prediabetes, meaning that their blood glucose levels are higher than normal but below the diabetes range [23]. Moreover, one study has projected the incidence of diabetes to be about 15 cases per 1,000 in 2050 compared to 8 cases per 1,000 in 2008 (essentially a doubling, in other words) [24]. Hence, to the extent that hospitalizations can be prevented by more effective pharmacotherapy in the future, there will be substantial potential cost savings available.

### Consistency with prior work

The conservative readmission rates (approximately 15 %–25 %) in our study, relative to those mentioned in the introduction, probably result from the omission of insulin users, who are likely to be patients with more comorbidities. Ng and colleagues found that, for a Canadian cohort of patients with type 2 diabetes, those taking insulin had a significantly higher likelihood of a hospitalization (OR = 1.7, 95 % CI: 1.4–2.0), controlling for numerous demographic, socioeconomic, and health-status characteristics [25].

Our study’s results are consistent with research that has compared the antidiabetic drugs with respect to outcomes that could lead to hospitalizations. Bodmer and colleagues found that use of SUs was associated with a 2.8 times higher risk of hypoglycemia than metformin. [26] Evans and colleagues found that patients treated with SUs only were at higher risk of adverse cardiovascular outcomes than those treated with metformin alone [27]. Eurich and colleagues found that, compared with SU monotherapy, metformin, alone or in combination, was associated with a statistically significant lower morbidity and mortality for patients suffering from both type 2 diabetes and heart failure [28]. Finally, Horsdal and colleagues found lower 30-day mortality rates among users of metformin (HR 0.32, 95 % CI: 0.15–0.68) as well as patients without pharmacotherapy (HR 0.58, 95 % CI: 0.36–0.93) compared with users of SUs [29].

In this study, the patients in the SU cohort were on average 8 years older than those taking a non-SU AHA, a fact that helps to explain the difference in readmission cost between the two cohorts. Older patients were both more likely to have additional comorbidities, requiring attention during the hospitalization, as well as more advanced diabetes. Moreover, as seen in Table 2, the SU cohort, before propensity-score adjustment, had a significantly higher percentage of patients with fair or poor perceived health status than those in the other cohort of patients, potentially lengthening and/or complicating their hospital stays. This result is consistent with that of Raebel and colleagues who found that older patients as well as those with elevated serum creatinine were more likely to initiate SU therapy than younger, less severe patients [30]. This result, however, is not consistent with that of Desai and colleagues who found that older patients (70 years old and older) were significantly more likely to receive metformin (not an SU) as initial therapy than those younger than 70 [31].

Unmarried patients had a statistically significant higher risk of hospital readmission than patients who were married. This result is consistent with a number of studies showing that marriage has a protective effect on mortality and hospitalization [32, 33]. The statistically significant effect of eye disease makes sense given that the strongest predictor for development and progression of retinopathy is duration of diabetes [34]. Patients suffering diabetes for longer are more likely to develop eye problems as well as other complications leading to hospitalizations.

### Hospital readmission trends in the 2000s

There was a significant fall in the number of multiple hospitalizations for patients with diabetes over time. Readmissions were less likely to occur later in the study period than earlier. This result is consistent with what Cunningham and Carrier found in their study of the trend in medical-care costs for nonelderly adults with diabetes [35].

### Pharmacotherapy trends in the 2000s

The treatment of diabetes changed substantially over the decade of the 2000s. Specifically, there was a de-emphasis on the use of the SU drug class. From the MEPS data (Table 2), the share of SU patients was higher in the earliest time period than the latest, while the share of noSU patients was higher in the latest period. Consistent with our results, Desai and colleagues found that the proportion of patients initially treated with an SU decreased from 2006 to 2008. Over the same period, there was a significant increase in the use of metformin and DPP-4 inhibitors and a significant decline in the use of TZDs [31]. A longer trend analysis was undertaken by Alexander and colleagues. In their study, SU utilization (monotherapy or combined) decreased from 67 % of treatment visits in 1994 to 34 % in 2007 [36]. A new class of antidiabetic agents for the treatment of type 2 diabetes, the sodium-glucose co-transporter 2 (SGLT2) inhibitors, has become available since the end of our study period. Canagliflozin (Invocana®), dapagliflozin (Forxiga®), and empagliflozin (Jardiance®) were approved by the Food and Drug Administration in April 2013, January 2014, and August 2014, respectively [37–39]. Additional treatment options may encourage a decline in the use of older medications.

In the 2015 position statement (2015 diabetes guidelines) of the American Diabetes Association, the suggested approach to the management of hyperglycemia in most individuals with type 2 diabetes includes intervention at the time of diagnosis with metformin (the only marketed biguanide in the U.S.) in combination with lifestyle changes [9]. If noninsulin monotherapy at maximal tolerated dose is not effective within 3 to 6 months, a second oral agent or insulin should be added as a means of achieving and maintaining recommended levels of glycemic control [9]. For some older adults, however, metformin may be contraindicated because of renal insufficiency or heart failure. Moreover, again a concern primarily for older adults, TZDs may cause fluid retention, which may lead to heart failure [9]. For these patients, the use of alternative AHAs, including SUs, may be warranted. Because our study period ended in 2010, healthcare providers did not have access to the 2015 guidelines. Earlier position statements (see Nathan and colleagues [40, 41]), however, provided pharmacotherapy guidance for physicians. They also suggested reduced reliance on the SUs.

### Limitations

The MEPS has been widely used in the study of diabetes and has served as the database of choice to examine many aspects of this chronic condition. However, the lack of clinical data on disease severity (there are no Hba1c values, for example) and progression limits our ability to estimate causal relationships between drug use and risk of hospital readmission. To the extent that unobserved variables were associated with the propensity to receive SU treatment, our results may be biased. Additionally, we cannot rule out the possibility that differences in readmission rates may be related to beneficial effects of other AHAs, especially those of metformin, rather than detrimental effects of SUs. Because of the limited longitudinal nature of the MEPS database (as opposed to claims databases), we were unable to identify (1) patients who were initiating antidiabetic pharmacotherapy; (2) patients’ incident hospitalizations, implying that their first hospitalizations may themselves have been readmissions; or (3) patients’ prior healthcare utilization, which could be predictive of future utilization, including hospitalizations. Furthermore, we defined multiple-hospitalization patients as those who had two or more hospital admissions during a year’s time. Whereas previous literature has looked specifically at time between discharge and readmission [5, 6], the MEPS data were not complete enough in many cases to measure time between hospitalizations that precisely.

## Conclusions

Our study suggests that among patients with type 2 diabetes, SU use is associated with an approximately 30 % increased risk for hospital readmission and 45 % higher readmission costs per readmission compared to other oral AHAs. Because inpatient hospitalizations represent the largest share of the substantial economic burden of diabetes in the United States, reducing preventable hospitalizations, especially readmissions, will help to reduce direct healthcare costs.

## Appendix 1

**Table 4:** ICD-9 Codes for diabetes-related conditions

Condition	ICD-9 code(s)
Diabetes	250
Cardiovascular disease	410–414, V458, 398, 428,426,427,785, V450, V533, 430–438, 437, 415–417, 440–414
Renal disease	585–586, V420, V451, V56, 580–584, 590, 595,597, 598, 599
Lower extremity disease	337,342–344, 354,355,356,357,358, 440, 442, 443, 444, 445, 711, 718, 727, 739, 735, 736, 784, 020, 021, 022, 031, 032, 035, 039, 680–682, 684–686, 690, 694–698, 700–703, 707, 709, V133, V423
Eye disease	361,362, 365–369,V431, V410
Mycoses	110–112, 114, 115, 116–118
Fluid and electrolyte disorders	276
Fractures	800–829
Syncope	780
Other injuries	959
Hypoglycemia	251
Other disorders of soft tissue	729

**Table 5:** Medication classification codes for oral antihyperglycemic agents

Drug class	Therapeutic classification code
Sulfonylureas	213
Other oral antihyperglycemic agents	
Biguanides	214
Thiazolidinediones	271
Alpha-glucosidase inhibitors	216
Meglitinides	282
Dipeptidyl peptidase 4 inhibitors	371
Antidiabetic combinations	314

## Abbreviations

AHA: Antihyperglycemic agent
CI: Confidence interval
DPP-4: Dipeptidyl peptidase 4
HbA1c: Glycated hemoglobin (A1c)
HR: Hazard ratio
ICD-9: International classification of diseases, Ninth revision
MEPS: Medical expenditure panel survey
OR: Odds ratio
SGLT2: Sodium-glucose co-transporter 2
SU: Sulfonylurea
TZD: Thiazolidinedione
